# Diagnostic accuracy of salivary gland ultrasonography with different scoring systems in Sjögren’s syndrome: a systematic review and meta-analysis

**DOI:** 10.1038/s41598-018-35288-5

**Published:** 2018-11-20

**Authors:** Mingzhu Zhou, Shuju Song, Shanshan Wu, Ting Duan, Letian Chen, Jingyi Ye, Jun Xiao

**Affiliations:** 1 Department of Rheumatology and Immunology, Beijing Friendship Hospital, Capital Medical University, Beijing, P. R. China; 2 Department of National Clinical Center of Digestive Diseases, Beijing Friendship Hospital, Capital Medical University, Beijing, P. R. China; 3 The Macrohard Institute of Health, Michigan, MI 48148 USA

## Abstract

Noninvasive objective salivary gland ultrasonography (SGU) had been widely used to evaluate major salivary gland involvement in primary Sjögren’s syndrome (pSS) and treatment responses. However, the evaluation score, diagnostic sensitivity, and diagnostic specificity significantly varied among clinical studies. We conducted this meta-analysis to assess the diagnostic accuracy of different SGU scoring systems using the American-European Consensus Group criteria. Of the 1301 articles retrieved from six databases, 24 met the criteria for quality assessment and 14 for meta-analyses. The pooled sensitivities were 75% (0–4) with I^2^ = 92.0%, 84% (0–16) with I^2^ = 63.6%, and 75% (0–48) with I^2^ = 90.9%; the pooled specificities were 93% (0–4) with I^2^ = 71.5%, 88% (0–16) with I^2^ = 65.4%, and 95% (0–48) with I^2^ = 83.9%; the pooled diagnostic odds ratios were 71.26 (0–4) with I^2^ = 0%, 46.3 (0–16) with I^2^ = 73.8%, and 66.07 (0–48) I^2^ = 0%; the areas under the SROC curves were 0.95 (0–4), 0.93 (0–16), and 0.94 (0–48). These results indicated that the 0–4 scoring system has a higher specificity and a less heterogeneity than other systems, and could be used as a universal SGU diagnostic standard.

## Introduction

Sjögren’s syndrome (SS) is the second most prevalent autoimmune rheumatic disease with a prevalence of 0.05%^1,2^. SS can affect any body organ or system such as interstitial lung disease, pulmonary hypertension, amyloidosis, and mucosa-associated lymphoid tissue lymphoma^3–6^. SS patients constantly suffer dry mouths, dry eyes, dry skin, a chronic cough, vaginal dryness, numbness in the arms and legs, feeling tired, muscle and joint pains, and thyroid problems.

The pathophysiology of SS has not been fully understood. It is believed to involve a combination of genetics and an environmental trigger such as exposure to a virus or bacteria^7,8^. Due to the vast range of SS symptoms and the similarity between symptoms of SS and those of other conditions, diagnosis of SS is complicated and difficult. Also, since the SS symptoms such as dry eyes and dry mouth are very common, especially among patients over 40 years old, it is often mistaken as age-related, thus ignored. In addition, some medications can also cause symptoms similar to those of this autoimmune disorder. These unspecific and common symptoms make the objective diagnosis crucial.

Sialography and biopsy of the labial minor salivary glands (“lip biopsy”) are the established and objective examinations in diagnosing SS. However, the invasiveness and the complications from these procedures limit their clinical uses. Recently, noninvasive objective salivary gland ultrasonography (SGU) had been widely used to evaluate major salivary gland involvement in primary SS (pSS) and treatment responses^9^. Plenty of clinical studies demonstrated that SGU is sensitive and specific to pSS^10–33^. Some studies showed that the results from SGU were highly consistent to those from Sialography and lip biopsy^16,17,23^. It was recommend that SGU be used as a SS diagnostic tool^24^. However, the evaluation score, diagnostic sensitivity, and diagnostic specificity significantly varied among these clinical studies^10–33^. Therefore, a meta-analysis of these exiting clinical studies is needed to evaluate which scoring system has lower heterogeneity.

To our knowledge, the meta-analysis conducted by Delli *et al*. is the only meta-analysis that assessed the diagnostic properties of SGU in the diagnosis of SS^2^. It has been established that a single gold standard should be used in meta-analysis. However, multiple gold standards i.e., FC, JDC, CC, TC, ECSG, AECG, RJDC, were used by Delli *et al*.^2^. In addition, Delli *et al*. did not performed subgroup analysis, likely introducing bias. Therefore, a meta-analysis of these exiting studies by subgroups using a single gold standard is urgently needed to recommend a guideline regarding whether SGU is a highly specific pSS diagnostic tool and which SGU scoring system can be used as an universal diagnostic standard. To that end, we used the American-European Consensus Group (AECG) criteria as the gold standard and performed subgroup analyses per SGU scoring system.

## Results

### Study identification and selection

**Figure 1:**
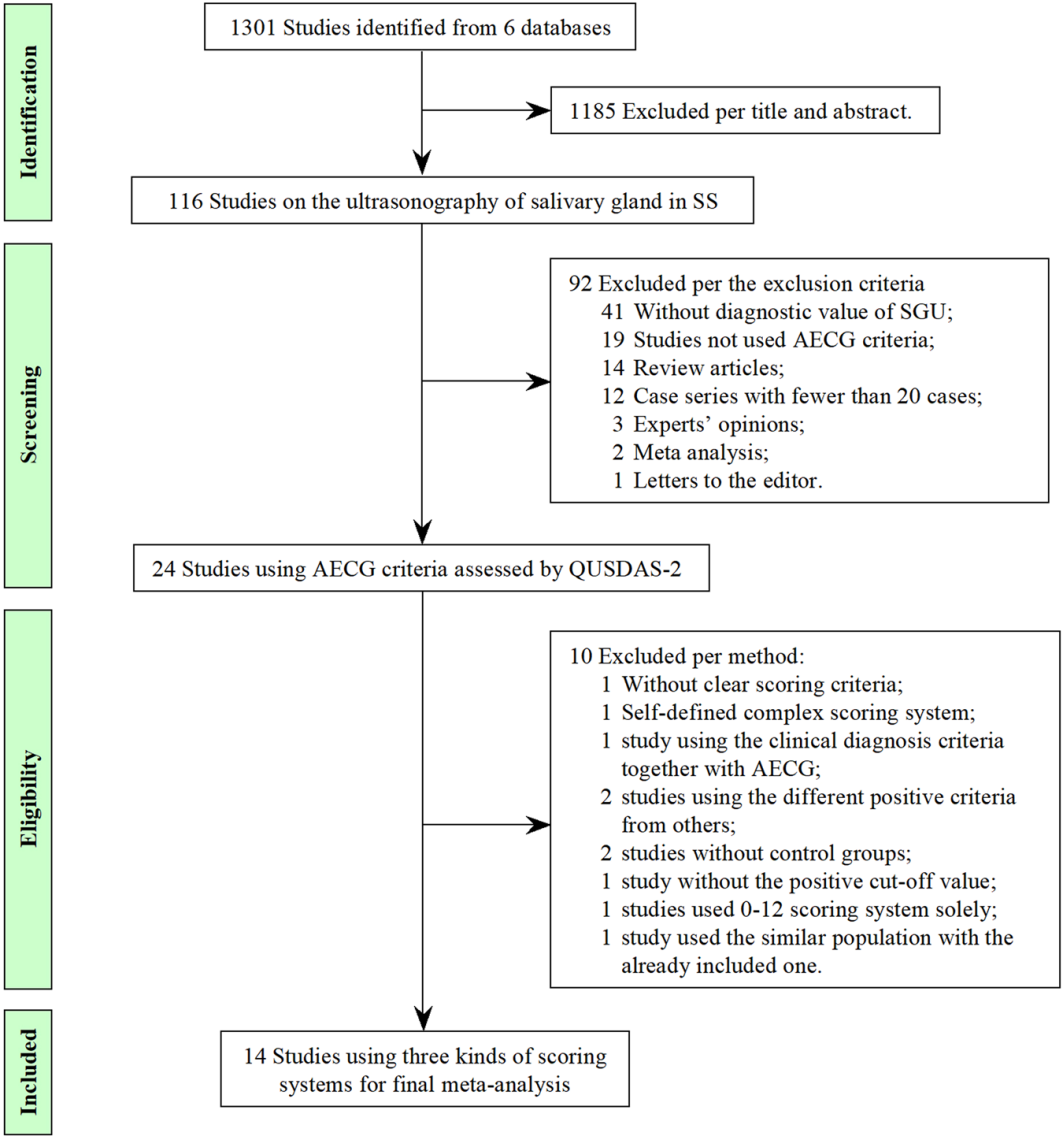
The flowchart of the studies included in this meta-analysis.

A total of 1301 studies were identified in the six databases. One thousand one hundreds and eighty-five studies were excluded per titles and abstracts; 92 per the exclusion criteria. The quality assessment was performed using QUSDAS-2 in the remaining 24 studies (Fig. 1), all of which used the AECG criteria for diagnosis of SS. Of the 24 studies, one study didn’t report about the scoring system clearly^17^, one used a self-defined complex scoring system^21^. Four scoring systems were used in 22 studies. Because 0–12 scoring system was used in only two studies, the final meta-analysis focused on the included 14 studies with three scoring systems as subgroups (0–4, 0–16, and 0–48).

### Study characteristics

A total of 3360 patients were enrolled in the 24 studies, including 1976 SS patients and 1384 control subjects (Table 1). Fifteen studies only included pSS patients, 3 studies included both pSS and secondary SS (sSS) patients, and 6 studies didn’t specify the type of the disease (pSS or sSS); 4 studies used sSS patients as control, 18 studies used subjects with sicca symptoms but not SS as control, 12 studies used healthy controls as control. Overall, 12 studies had only one control group; 10 studies had more than one control groups; and 2 studies had no control group.

**Table 1:** Characteristics of the 24 included studies (AECG as the diagnostic criteria).

Study	Country	Age range	Male n (%)	Study design	Total pt #	# of SS		# of controls			Scoring system	+score of US
						pSS	sSS	sSS	Sicca	HC		
El Miedany *et al*.^10^	Egypt	47–66	9 (19%)	cross-sectional cohort	87	47			20	20	0–3	≥1
Niemela *et al*.^11^	Finland	18–67	1 (4%)	cross-sectional cohort	81	27			27	27	0–4	≥1
Su *et al*.^12^	China	44–64	not specified	case-control	63	28			5	30	0–4	≥1
Hocevar *et al*.^13^	Slovenia	not specified	not specified	prospective cohort	218	68			150		0–48	≥17
Yang *et al*.^14^	China	20–58	not specified	retrospecctive study	41	41					0–4	≥2
Song *et al*.^15^	China	26–65	12 (12%)	case-control	128	98				30	0–4	≥1
Salaffi *et al*.^16^	Italy	30–78	3 (4%)	prospective cohort	156	77			79		0–16	≥6
Poul *et al*.^17^	UK	20–85	5 (14%)	prospective cohort	60	36	9		15		unknown	
Milic *et al*.^18^	Serbia	21–78	4 (4%)	prospective cohort	135	107			28		0–48	≥19
Milic *et al*.^19^	Serbia	21–78	6 (5%)	prospective cohort	245	115		44	50	36	0–12	≥6
Xu *et al*.^20^	China	28–78	0 (0%)	case-control	103	44			27	32	0–16	≥8
Takagi *et al*.^21^	Japan	56 ± 13	20 (11%)	prospective cohort	360	134	54		172		0–4	≥1
Kong *et al*.^22^	China	27–63	6 (11%)	case-control	84	15	39			30	0–48	unknown
Milic *et al*.^23^	Serbia	21–78	10 (7%)	prospective cohort	190	140			50		0–16	≥7
Cornec *et al*.^24^	France	56.8 ± 12.7	7 (9%)	prospective cohort	158	78			80		0–4	≥2
Theander *et al*.^25^	Sweden	20–91	not specified	cross-sectional cohort	162	105		6	19	32	0–3	≥2
Hammenfors *et al*.^26^	Norway	not specified	6 (6%)	cross-sectional cohort	97	97					0–3	≥2
Baldini *et al*.^27^	Italy	47 ± 13	2 (4%)	cross-sectional cohort	107	50			57		0–3	≥2
Zhang *et al*.^28^	China	56.42 ± 10.21	4 (4%)	prospective cohort	162	105			41	16	0–16	≥7
											0–48	≥15
Lin *et al*.^29^	China	46.3 ± 13.1	6 (14)	prospective cohort	94	44		14	36		0–12	≥6
											0–16	≥6
											0–48	≥17
Zhou *et al*.^30^	China	32–80	1 (2%)	case-control	85	53				32	0–4	≥2
Zhou *et al*.^31^	China	32–82	2 (3%)	case-control	165	71		45		49	0–4	≥2
Chen *et al*.^32^	China	23–77	1 (2%)	cross-sectional cohort	136	51			35	50	0–3	≥1
Qi *et al*.^33^	China	49.75 ± 15.52	8 (6%)	retrospective cohort	243	134			109		0–3	≥2
											0–16	≥5

PSS = primary Sjögren’s syndrome; pt = patient; sSS = second Sjögren’s syndrome; HC = healthy control; US = ultrasonography.

### Ultrasonography scoring systems and the subgroups

Fourteen studies used 0–4 scoring system including 0–3 scoring system (Table 1)^34^. The positive criteria is mild parenchymal inhomogeneity seen as multiple hypoechogenic areas measuring <2 mm with blurred borders. Eight Studies used the scores 0–4 for counting. Six studies used the scores 0–3 for counting (the positive criteria was mentioned above). Seven studies were excluded including one study using AECG criteria partly as gold standard^24^, two using the positive criteria lower than the above-mentioned criteria^12,15^, two having no control groups^14,26^, one using a self-defined complicated scoring system^21^, and one using the same patient population^30^. The remaining seven studies were included as the 0–4 scoring subgroup in the meta-analysis^10,11,25,27,31–33^.

Two studies used 0–12 scoring system^19,29^. The scores ranged from 0 to 12, and ≥6 score was considered as positive criteria. The 0–12 scoring system was excluded from meta-analysis due to small sampling.

Six studies used 0–16 scoring system, which was first reported by Salaffi *et al*.^35^. The scores ranged from 0 to 16. One study considered ≥5 as positive criteria^33^; two studies ≥6^16,29^; two studies ≥7^23,28^; and one study ≥8^20^. These six studies included as the 0–16 scoring subgroup in the meta-analysis.

Five studies used 0–48 scoring system, which was first reported by Hocevar *et al*.^13^. The scores ranged from 0 to 48. Two studies considered ≥17 as positive criteria^13,29^; one study ≥15^28^; one study ≥19^18^; and one study didn’t describe the cut-off value, which was excluded^22^. The four studies, which described the cuff-off values, were included as the 0–48 scoring subgroup in the meta-analysis.

Taken all together, 14 studies of the three subgroups were included in the meta-analysis, including 11 studies used only one scoring system, 2 studies used two scoring systems, and 1 study used three scoring systems (Tables 1 and 2).

**Table 2:** The sensitivity, specificity, and diagnostic OR of the three scoring systems (AECG as the diagnostic criteria).

	Cut-off value	Sensitivity (95% Cl)	Specificity (95% Cl)	Diagnostic OR (95%Cl)
**0–4scoring system**		**0**.**75** (**0**.**71–0**.**79**)	**0**.**93** (**0**.**90–0**.**95**)	**71**.**26** (**42**.**29–120**.**09**)
El Miedany *et al*.^10^	≥1	0.94 (0.82–0.99)	0.95 (0.83–0.99)	278.67 (44.21–1756.56)
Niemela *et al*.^2^	≥1	0.78 (0.58–0.91)	0.94 (0.85–0.99)	59.50 (13.60–260.37)
Theander *et al*.^25^	≥2	0.52 (0.42–0.62)	0.98 (0.91–1.00)	61.60 (8.22–461.650)
Baldini *et al*.^27^	≥2	0.66 (0.51–0.79)	0.98 (0.91–1.00)	108.71 (13.83–854.74)
Zhou *et al*.^31^	≥2	0.62 (0.50–0.73)	0.98 (0.89–1.00)	78.22 (10.20–600.03)
Chen *et al*.^32^	≥1	0.92 (0.81–0.98)	0.92 (0.81–0.98)	135.13 (31.88–572.78)
Qi *et al*.^33^	≥2	0.90 (0.84–0.95)	0.83 (0.75–0.90)	47.06 (21.93–100.97)
**0–16 scoring system**		**0**.**84** (**0**.**81–0**.**87**)	**0**.**88** (**0**.**85–0**.**91**)	**46**.**3** (**19**.**95–107**.**44**)
Salaffi *et al*.^16^	≥6	0.75 (0.64–0.84)	0.84 (0.74–0.91)	15.50 (7.04–34.11)
Xu *et al*.^20^	≥8	0.93 (0.81–0.99)	0.97 (0.88–1.00)	389.50 (62.25–2437.01)
Milic *et al*.^23^	≥7	0.86 (0.79–0.91)	0.94 (0.83–0.99)	94.00 (26.68–331.22)
Zhang *et al*.^28^	≥7	0.80 (0.71–0.87)	0.93 (0.83–0.98)	53.00 (17.24–162.95)
Lin *et al*.^29^	≥6	0.80 (0.65–0.90)	0.78 (0.64–0.88)	13.79 (5.11–37.19)
Qi *et al*.^33^	≥5	0.90 (0.84–0.95)	0.87 (0.79–0.93)	63.16 (28.34–140.75)
**0–48 scoring system**		**0**.**75** (**0**.**70–0**.**80**)	**0**.**95** (**0**.**91–0**.**97**)	**66**.**07** (**33**.**73–129**.**42**)
Hocevar *et al*.^13^	≥17	0.59 (0.46–0.71)	0.99 (0.95–1.00)	105.71 (24.15–462.76)
Milic *et al*.^18^	≥19	0.65 (0.56–0.74)	1.00 (0.88–1.00)	107.16 (6.36–1804.92)
Zhang *et al*.^28^	≥15	0.89 (0.81–0.94)	0.84 (0.72–0.93)	41.33 (16.28–104.95)
Lin *et al*.^29^	≥17	0.91 (0.78–0.97)	0.92 (0.81–0.98)	115.00 (27.00–489.88)

OR = odd ratio.

### Diagnostic properties

The diagnostic properties of ultrasonography were compared among the 14 studies using the AECG criteria (Table 2). In particular, for the 14 studies included in our meta-analysis, the cut-off values ranged 1–2 (0–4 scoring system), 5–8 (0–16 scoring system), and 15–19 (0–48 scoring system), respectively. The sensitivity ranged 52–94% (0–4 scoring system), 75–93% (0–16 scoring system), and 59–91% (0–48 scoring system), respectively. The diagnostic specificity ranged 83–98% (0–4 scoring system), 78–97% (0–16 scoring system), and 84–100% (0–48 scoring system), respectively. The diagnostic OR ranged 47.06–278.67 (0–4 scoring system), 13.79–389.50 (0–16 scoring system), and 41.33–115.00 (0–48 scoring system), respectively.

The 0–4 scoring system has the least variations in specificity and diagnostic OR (0.90–0.95 and 42.29–120.09, respectively) when compared with the 0–16 scoring system (0.85–0.91 and 19.95–107.44, respectively) and the 0–48 scoring system (0.91–0.97 and 33.73–129.42, respectively) while the three systems have similar variations in sensitivity (0.71–0.79 in 0–4, 0.81–0.87 in 0–16, 0.70–0.80 in 0–48). In addition, the 0–4 scoring system had a universal cut-off value of 1 or 2 while the other two scoring systems did not. These results indicated that 0–4 scoring system is a more consistent scoring system.

### Diagnostic accuracy

In the 0–4 scoring system for sensitivity, the I^2^ index was 92.0%, (df = 6, p < 0.001) with a pooled sensitivity was 75% (71–79%) (Table 3); for specificity, the I^2^ index was 71.5%, (df = 6, p = 0.0018) with a pooled specificity was 93% (90–95%); for DOR, the I^2^ index was 0%, (df = 6, p = 0.643) with the pooled DOR was 71.26 (42.29–120.09). In the 0–16 scoring system for sensitivity, the I^2^ index was 63.6%, (df = 5, p = 0.0174) with a pooled sensitivity was 84% (81–87%); for specificity, the I^2^ index was 65.4%, (df = 5, p = 0.0129) with a pooled specificity was 88% (85–91%); for DOR, the I^2^ index was 73.8%, (df = 5, p = 0.0019) with the pooled DOR was 46.3 (19.95–107.44). In the 0–48 scoring system for sensitivity, the I^2^ index was 90.9%, (df = 3, p < 0.001) with a pooled sensitivity was 75% (70–80%); for specificity, the I^2^ index was 83.9%, (df = 3, p = 0.0003) with a pooled specificity was 95% (91–97%); for DOR, the I^2^ index was 0%, (df = 3, p = 0.551) with the pooled DOR was 66.07 (33.73–129.42). In summary, 0–16 scoring system had the highest sensitivity (84%) with relatively small I^2^ (63.6%); 0–48 scoring system had the highest specificity (95%), which was similar to that of 0–4 (93%); 0–4 and 0–48 scoring systems had the best DOR (I^2^ = 0%).

**Table 3:** The meta-analysis results of three scoring systems (AECG as the diagnostic criteria).

	Scoring System		
	0–4	0–16	0–48
Sensitivity			
Pooled Sensitivity (95% CI)	0.75 (0.71–0.79)	0.84 (0.81–0.87)	0.75 (0.70–0.80)
Chi-square (Degree of Freedom)	74.65 (6)	13.74 (5)	32.83 (3)
P Value	0.0000	0.0174	0.0000
Inconsistency (I^2^)	92.0%	63.6%	90.9%
Specificity			
Pooled Specificity (95% CI)	0.93 (0.90–0.95)	0.88 (0.85–0.91)	0.95 (0.91–0.97)
Chi-square (Degree of Freedom)	21.04 (6)	14.47 (5)	18.69 (3)
P Value	0.0018	0.0129	0.0003
Inconsistency (I^2^)	71.5%	65.4%	83.9%
Diagnostic Odds Ratio			
Pooled Diagnostic Odds Ratio (95% CI)	71.26 (42.29–120.09)	46.3 (19.95–107.44)	66.07 (33.73–129.42)
Cochran-Q (Degree of Freedom)	4.25 (6)	19.07 (5)	2.11 (3)
P Value	0.6430	0.0019	0.5507
Inconsistency (I^2^)	0.0%	73.8%	0.0%
Tau-squared	0.0000	0.7812	0.0000

Cl = confidence interval.

**Figure 2:**
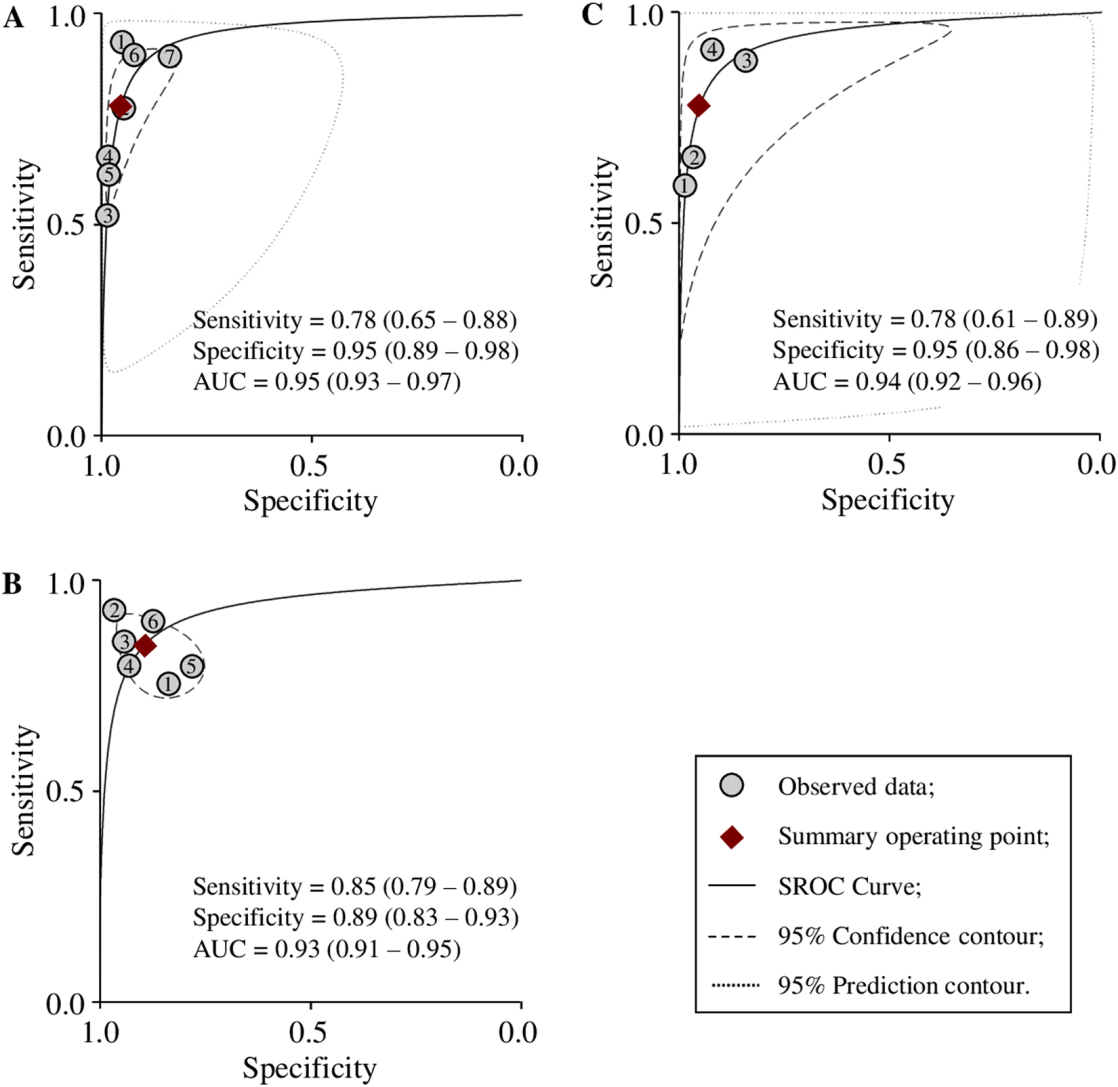
SROC curves of 0–4 (**A**) 0–16 (**B**) and 0–48 (**C**) scoring systems.

Due to the different cut-off values of the scoring systems, SROC analyses were performed (Fig. 2). The summary operating sensitivities were 78% (65–88%) (0–4 scoring system), 85% (79–89%) (0–16 scoring system), and 78% (61–89%) (0–48 scoring system), respectively; the summary operating specificities were 95% (89–98%) (0–4), 89% (83–93%) (0–16), and 95% (86–98%) (0–48), respectively; the areas under curves (AUC) were 0.95 (0.93–0.97) (0–4), 0.93 (0.91–0.95) (0–16), and 0.94 (0.92–0.96) (0–48), respectively, indicating that these three systems were accurate diagnostic systems.

Taken all together, the heterogeneities of the pooled DOR for 0–4 and 0–48 scoring systems was 0%, or no heterogeneities, suggesting that these two scoring systems be reliable. However, it seemed that the 0–4 scoring system was the best among the three scoring systems because (i) the cut-off value was pre-specified in the 0–4 scoring system while the cut-off values in both 0–16 and 0–48 scoring system were different among the studies, and (ii) the 0–4 scoring system has the least variations in specificity and diagnostic OR (0.90–0.95 and 42.29–120.09, respectively) when compared with the 0–16 scoring system (0.85–0.91 and 19.95–107.44, respectively) and the 0–48 scoring system (0.91–0.97 and 33.73–129.42, respectively).

### Quality assessment and risk of bias of the studies

**Figure 3:**
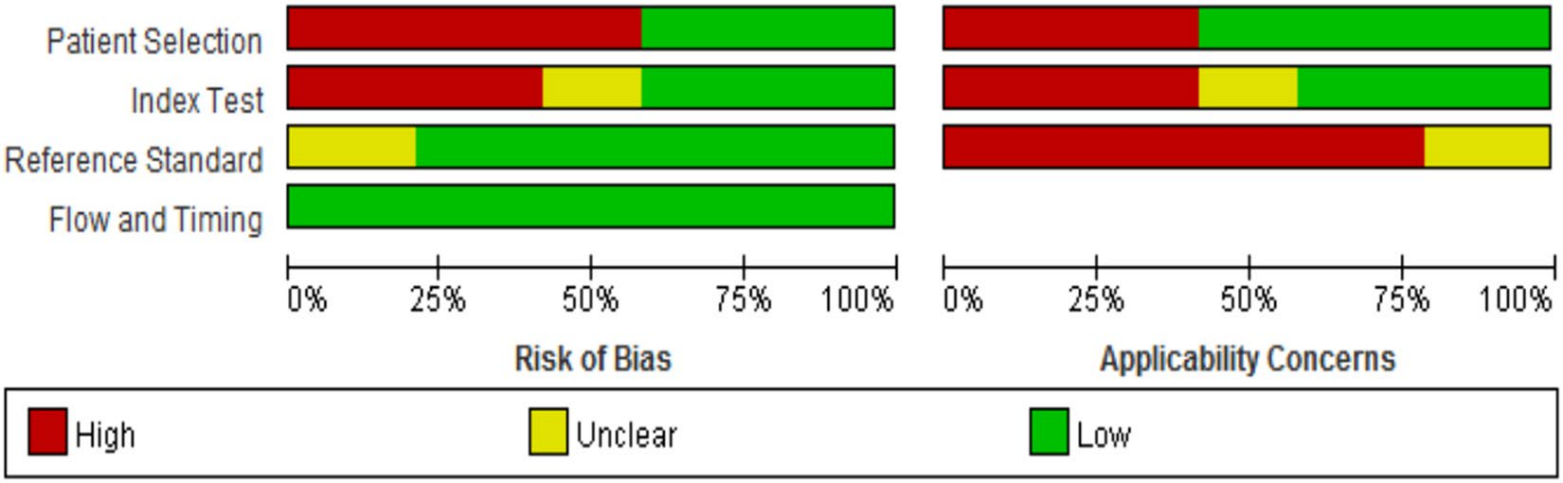
Percentages of studies in the QUADAS-2 analysis for the items of risk of bias and applicability Concerns.

High risk of bias was observed in “patient selection” due to the variations of inclusion and exclusion criteria (e.g., whether a case-control study was included or excluded) as well as the patient selection criteria (i.e., whether patients were enrolled consecutively or randomly) (Fig. 3). High risk of bias was also observed in the “conduct and interpretation of index test” due to the designs of the original studies (e.g., whether the SGU results were interpreted with the knowledge of the SS diagnosis; whether a threshold was pre-specified).

Twenty-four studies were included in the QUADAS-2 quality assessment, including 21 studies used only one scoring system, 2 studies used two scoring systems, and 1 study used three scoring systems (Table 1). The most frequent high risks of bias were biases due to patient selection and index test. In particular, 14 (58.3%) studies and 10 (41.7%) studies were rated as “high risk” in terms of the biases due to patient selection and due to index test (Table 4). In contrast, all the studies were rated as “low risk” in terms of the biases due to reference standard and due to flow and timing. More studies had low concerns in the applicability of patient selection (58.3%) than in the applicability of index test (41.7%) and the applicability of reference standard (0%). These results indicate that the applicability of SGU was high.

**Table 4:** Risk of bias and applicability of the studies included.

	Risk of bias				Concerns about applicability		
	Bias due to patient selection	Bias due to index test	Bias due to reference standard	Bias due to flow and timing	Applicability of patient selection	Applicability of index test	Applicability of reference standard
El Miedany *et al*.^10^	High risk	Low risk	Low risk	Low risk	Low concern	High concern	Low concern
Niemela *et al*.^11^	High risk	Low risk	Low risk	Low risk	Low concern	High concern	Low concern
Su *et al*.^12^	High risk	Unclear	Low risk	Low risk	Low concern	Unclear	Low concern
Hocevar *et al*.^13^	Low risk	High risk	Low risk	Low risk	High concern	Low concern	Low concern
Yang *et al*.^14^	High risk	Unclear	Low risk	Low risk	Low concern	Unclear	Low concern
Song *et al*.^15^	High risk	Unclear	Low risk	Low risk	Low concern	Unclear	Low concern
Salaffi *et al*.^16^	Low risk	High risk	Unclear	Low risk	High concern	Low concern	Unclear
Poul *et al*.^17^	Low risk	Low risk	Unclear	Low risk	High concern	High concern	Unclear
Milic *et al*.^18^	Low risk	High risk	Low risk	Low risk	High concern	Low concern	High concern
Milic *et al*.^19^	Low risk	High risk	Unclear	Low risk	High concern	Low concern	Unclear
Xu *et al*.^20^	High risk	High risk	Low risk	Low risk	Low concern	Low concern	High concern
Takagi *et al*.^21^	Low risk	Low risk	Unclear	Low risk	High concern	High concern	Unclear
Kong *et al*.^22^	High risk	High risk	Low risk	Low risk	Low concern	Low concern	High concern
Milic *et al*.^23^	Low risk	High risk	Low risk	Low risk	High concern	Low concern	High concern
Cornec *et al*.^24^	Low risk	Low risk	Low risk	Low risk	High concern	High concern	High concern
Theander *et al*.^25^	High risk	High risk	Unclear	Low risk	Low concern	Low concern	Unclear
Hammenfors *et al*.^26^	High risk	Low risk	Low risk	Low risk	Low concern	High concern	High concern
Baldini *et al*.^27^	Low risk	Low risk	Low risk	Low risk	High concern	High concern	High concern
Zhang *et al*.^28^	High risk	High risk	Low risk	Low risk	Low concern	Low concern	High concern
Lin *et al*.^29^	Low risk	Low risk	Low risk	Low risk	High concern	High concern	High concern
Zhou *et al*.^30^	High risk	Low risk	Low risk	Low risk	Low concern	High concern	High concern
Zhou *et al*.^31^	High risk	Low risk	Low risk	Low risk	Low concern	High concern	High concern
Chen *et al*.^32^	High risk	Unclear	Low risk	Low risk	Low concern	Unclear	High concern
Qi *et al*.^33^	High risk	High risk	Low risk	Low risk	Low concern	Low concern	High concern

### An ultrasound picture scored with different scoring systems

**Figure 4:**
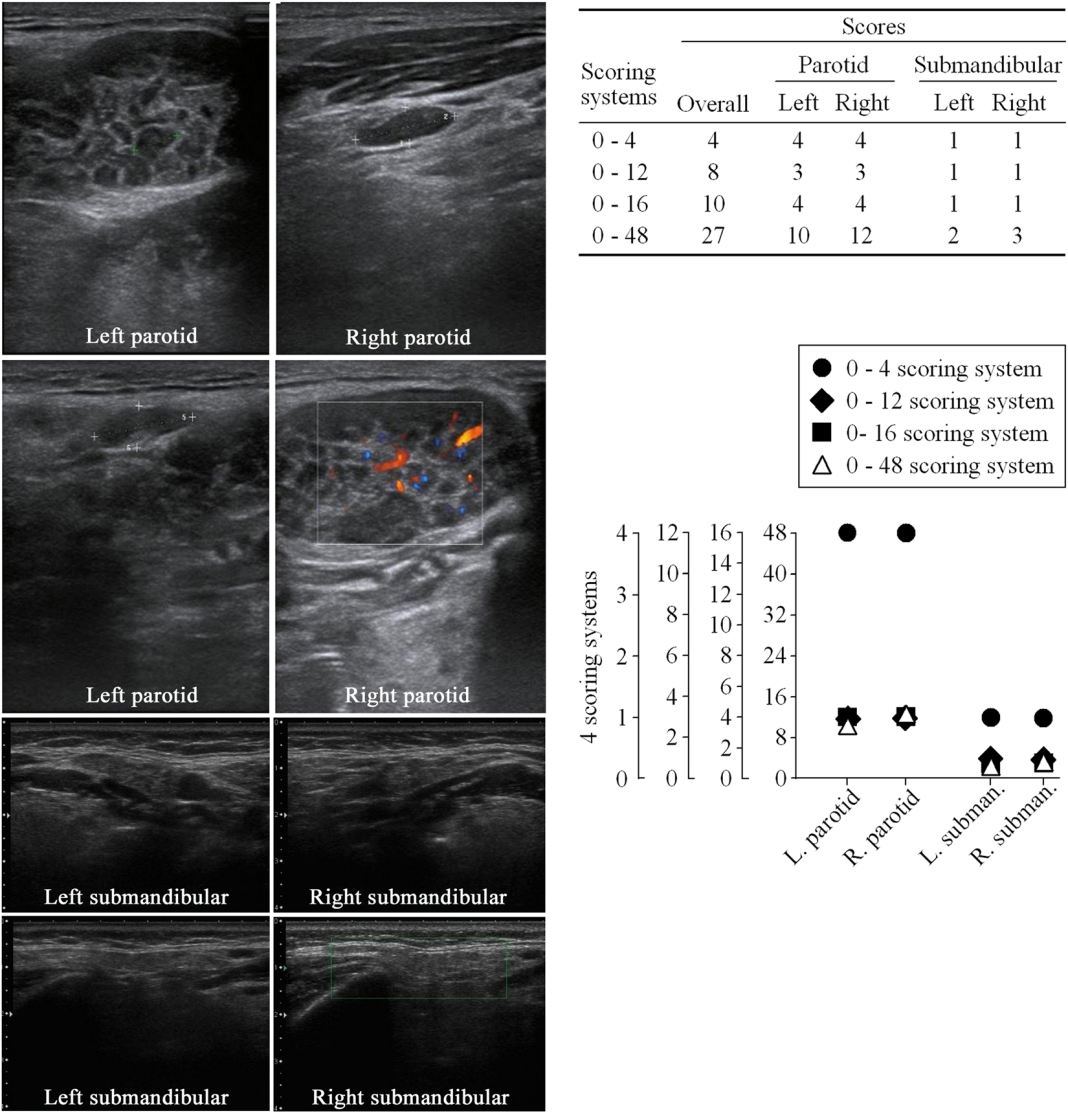
The ultrasound pictures of the parotid gland and submandibular gland from a patient (66 years old) diagnosed as Sjögren’s syndrome (left), the scores from different scoring systems (upper right), and direct comparison of different scoring systems (lower right).

Direct comparisons among different scoring systems on a same patient was performed (Fig. 4). 0–4 system is significantly distinguished from the other 3 systems while the 3 systems proportionally project among each other in essence (Fig. 4, lower right).

## Discussion

Plenty of clinical researches using different diagnostic criteria and scoring systems indicated that SGU is sensitive and specific to pSS. In contrast, the meta-analyses on these studies were scarce. Only two meta-analysis studies have published regarding the diagnostic value of SGU in SS patients^2,36^. One study compared the diagnostic properties of ultrasonography and sialography in SS, demonstrating that ultrasonography was comparable with sialography^36^. However, this meta-analysis only included six studies, and could not explain the diagnostic value of ultrasonography in SS. In addition, the assessment of research methodology was less rigorous, with high risk of bias in all QUADAS-2 domains, resulting in concerns regarding the outcomes^2^. The other study performed a good quality assessment^2^. However, this meta-analysis did not distinguish the diagnostic criteria and the scoring systems. In addition, the studies included were not rigidly designed and performed as their results showed significant heterogeneity. Therefore, quality of the pooled outcomes (sensitivity, specificity, and diagnostic odds ratio) was low. The likely source of this heterogeneity was the ultrasonography scoring systems. To our knowledge, the current study is the first meta-analysis to perform subgroup analyses regarding different scoring system using only one diagnostic criterion.

The different cut-off values in the 0–16 and 0–48 systems resulted in relative large heterogeneity of sensitivity and specificity. To decrease this heterogeneity, we conducted SROC curve analysis in the three subgroups. Our results indicted all the three systems are reliable diagnostic tools with similar accuracy (SROC AUC 0.95 (0–4), 0.93 (0–16), and 0.94 (0–48)).

In the 0–4 system, the sensitivity was 75%, specificity 93%, diagnostic DOR heterogeneity 0%, cut-off pre-specific. In addition, the operation was simple and the operation time was shorter. These advantages allowed the 0–4 system to outperform 0–16 and 0–48 systems. In contrast, although both 0–16 and 0–48 systems were reliable scoring systems with similar AUC, the cut-off values were not pre-specified indicating that these scoring systems could not be used as SGU diagnostic standard. Taken all together, the 0–4 scoring system seems to be a better scoring system being used as a universal SGU diagnostic standard with a higher specificity and a less heterogeneity than the other scoring systems (0–16 and 0–48). Actually, the 0–4 system is significantly distinguished from the other 3 systems (Fig. 4, lower right).

This study has several strengths. First, mainly four scoring systems are used clinically, each of which has it own advantages. It is of clinical significance to meta-analyze different scoring systems as subgroups, respectively, to decrease possible heterogeneity and establish which scoring system is overall the best. Our results indicted that the 0–4 scoring system was the best among the three scoring systems as the diagnostic criterion. In particular, the heterogeneity of the pooled DOR for 0–4 and 0–48 scoring systems was 0%, or no heterogeneity, indicating that these scoring systems are reliable. Between the two scoring systems, we think 0–4 scoring system is better, because the cut-off value is pre-specified. In contrast, the cut-off value of 0–48 scoring system is different among studies. In addition, the heterogeneity of the pooled specificity was high in 0–48 system. The heterogeneity of the pooled sensitivity of 0–4 and 0–48 scoring system was both very high. This might relate with the selection of patients and control groups.

Second, diagnostic criteria are clinically important as different criteria might result in different diagnosis. However, previous meta-analysis included studies using different diagnostic criteria, i.e., FC, JDC, CC, TC, ECSG, AECG, RJDC^2^. This meta-analysis only included studies that used AECG as a single criteria decreasing possible heterogeneity. Our results were consistent with clinical practice that AECG could be considered as an established diagnostic criteria for pSS.

Third, QUADAS-2 is the best quality assessment tool for diagnostic research. The quality of recent studies were higher than the past ones as showed by QUADAS-2^37^. In contrast to studies enrolled consecutive subjects or subjects with suspected SS, and divided into groups after the index and reference test^13,14,16–19,21,23,24,27,29^, this up-to-date meta-analysis included more recent rigidly designed studies and avoided the case-control type.

This study also has some limitations. First, the studies included varied in terms of (a) patient enrollment (random or not), which could cause the selection bias; (b) the blindness of the SGU examination to the diagnostic procedures, which could cause confirmatory bias (specifically, not blindness could cause confirmatory bias). This might be the major reason that the heterogeneity of sensitivity and specificity was high (I^2^ of the sensitivity is 92.0% (0–4), 63.6% (0–16), and 90.9% (0–48), respectively; I^2^ of the specificity is 71.5% (0–4), 65.4% (0–16), and 83.9% (0–48), respectively). SGU is highly specific in pSS. However, some studies only enrolled pSS patients while some enrolled both pSS and sSS patients. Regarding the control, it was ideal that control enrolls either sicca or healthy patients. However, some studies failed to do so directly resulting in the heterogeneity of sensitivity. In addition, the threshold of SGU was pre-specified only in 0–4 system while not pre-specified in other systems. However, these limitations unlikely could change our conclusion, which was drawn from the comparisons of subgroups.

Second, due to our rigid selection criteria, studies included in this meta-analysis used one SS diagnosis standard. However, two problems still existed: First, regarding the threshold of the SGU scores, the cut-off value was not pre-specified except in the 0–4 scoring systems; Second, most studies didn’t mention the interval between SGU and AECG diagnosis. Only in 7 studies that the SGU was performed simultaneously with the diagnostic procedures^13,16–18,23,27,29^. Regarding subgroup analysis, less than 8 studies were included in each subgroup so that the funnel plot is insignificant.

In conclusion, SGU is a highly specific pSS diagnostic tool. The 0–4 scoring system is a better scoring system as a universal SGU diagnostic standard in terms of specificity and heterogeneity than the other scoring systems (0–16 and 0–48).

## Methods

This review followed the guidelines of the Preferred Reporting Items for Systematic Reviews^38^ and Meta-Analysis and the Meta-Analysis of Observational Studies in Epidemiology^39^.

### Literature Search

Six databases (Embase, Pubmed, Cochrane library, China National Knowledge Infrastructure (CNKI), WanFang databases, and WeiPu Periodical Resource Integration Service Platform from September 1, 1982 until April 15, 2018) were searched with the keywords (“salivary gland”, “parotid gland”, or “submandibular gland”) and (“ultrasonography”, “ultrasound”, or “sonography”), and (“Sjögren’s syndrome”, “Sjögren syndrome”, “sicca syndrome”, or “sicca”).

### Study Selection

Inclusion criteria were studies containing data on the diagnostic value of SGU for pSS, using AECG criteria as the diagnostic criteria, and including more than 20 cases. Exclusion criteria for titles and abstracts included: case reports, case series with fewer than 20 cases, letters to the editor, experts’ opinions, review articles, studies without diagnostic value of SGU, and studies used non-AECG criteria as diagnostic criteria. The studies were fully assessed if the title and abstract only provided limited information or in case of doubt.

Two independent researchers (M.Z. and S.S.) initially evaluated the titles and abstracts for eligibility per inclusion and exclusion criteria. Disagreements were resolved through consensus. The full texts of eligible studies were screened by the diagnosis criteria of SS. The studies using the AECG criteria as the golden standard were finally selected for this study.

### Data Extraction

Two researchers (M.Z. and S.S.) extracted the data independently. Disagreements were resolved through consensus. Extracted information included description of population, publication year, study type, study design, diagnosis criteria for SS, the definition of the scoring systems in studies (Supplemental Materials STable 1), and ultrasonographic scoring system as well as true positive, true negative, false positive, and false negative.

### Quality Assessment

Two researchers (M.Z. and S.S.) assess the quality of the studies per QUADAS-2 (the revised Quality Assessment of Diagnostic Accuracy Studies) tool. Disagreements were resolved by discussion.

### Statistical Analysis

Selected studies were further divided into three subgroups, 0–4, 0–16, and 0–48 ultrasonographic scoring systems. The pooled diagnostic sensitivity, specificity, and odds ratio (DOR) were calculated for each subgroups. The heterogeneity of the pooled sensitivity, specificity, and DOR were measured by the inconsistency (I^2^) and Cochran Q test. The heterogeneity was a measure of the between-study variations and was used to assess whether the studies in a meta-analysis represented a single population or several different populations. The percentage measures of the heterogeneity among the enrolled articles were calculated as I^2^ index. Small heterogeneity in the enrolled articles was defined as I^2^ < 25%, moderate heterogeneity was defined as I^2^ 25–50%, obvious heterogeneity was defined as I^2^ > 50%. The Cochran Q test was used for calculating heterogeneity (P < 0.05). The random effects model was used for data analysis.

The risk of bias of the included studies was assessed by QUADAS-2 tool^37^. Quality assessment was performed with Review Manager software (version 5.3, The Nordic Cochrane Centre, The Cochrane Collaboration). Pooling of sensitivity, specificity, DOR, and heterogeneity test were performed with Meta-Disc software (version 1.4, Madrid, Spain). The summary receiver operating characteristic (SROC) curves were produced in STATA13.0.

## Electronic supplementary material

Supplementary materials

## Data Availability

The authors declare that the data in this research is available.
