# Supplementary material for: Diagnostic accuracy of salivary gland ultrasonography with different scoring systems in Sjögren’s syndrome: a systematic review and meta-analysis
Source: Sci Rep. 2018 Nov 20;8:17128. doi: 10.1038/s41598-018-35288-5 (PMC6244082; doi:10.1038/s41598-018-35288-5)
Supplement: Supplementary file 1 — Supplementary materials [file 41598_2018_35288_MOESM1_ESM.pdf]

Diagnostic accuracy of salivary gland ultrasonography with different scoring systems in Sjögren's syndrome: a systematic review and meta-analysis

Mingzhu Zhou[1], Shuju Song[1], Shanshan Wu[2], Ting Duan[1], Letian Chen[1], Jingyi Ye[3], and Jun Xiao[3,*]

1. Department of Rheumatology and Immunology, the Beijing Friendship Hospital affiliated to the Capital Medical University, Beijing, P. R. China
2. Department of National Clinical Center of Digestive Diseases, the Beijing Friendship Hospital affiliated to the Capital Medical University, Beijing, P. R. China
3. The Macrohard Institute of Health. Michigan, MI 48148, USA.

*: Corresponding Authors: Jun Xiao, PharmD., Ph.D. The Macrohard Institute of Health. 231 North Ave. Battle Creek, MI 49017. Email: jxiao@macrohardinstitute.org. Phone: (01) 269-224-3181. Fax: (01) 269-224-3180.

Stable 1. The scoring criteria of the four scoring systems

Reference	Ultrasonography (US) scoring system	Cutoff value
**0 - 4 scoring system**		
El Miedany et al. 2004.	Four grades of parenchymal inhomogeneity (PIH) were distinguished:<br>0: Normal homogenous parenchyma.<br>1: Mild PIH seen as diffuse hypoechoic areolae < 2 mm with blurred borders.<br>2: Moderate PIH seen as large hypoechoic areas, 2 – 6 mm in diameter, with sharp borders.<br>3: Severe PIH with > 6 mm circumscribed hypoechoic areas.<br>The overall score is the highest score in any of the 4 salivary glands.	1
Niemela et al. 2004.	The parenchymal structure of the glands was categorized into five stages (stage 1 - 4 were considered as a sign of primary SS):<br>0: Normal.<br>1: Mild PIH (hypoechoic areas < 2 mm).<br>2: Evident PIH (hypoechoic areas of 2 - 6 mm).<br>3: Gross PIH (hypoechoic areas > 6 mm).<br>4: Adipose degeneration of the gland (adipose tissue echogenicity and parenchymal atrophy).	1
Theander et al. 2014.	0: Parenchymal homogeneity.<br>1: Mildly PIH.<br>2: Several rounded hypoechoic lesions were present.<br>3: The rounded hypoechoic lesions were either numerous or confluent.<br>The overall score is the highest score in any of the 4 salivary glands.	2
Baldini et al. 2015.	1: A mild level of PIH, isolated hypoechoic areas.<br>2: An evident level of PIH, evident scattered hypoechoic areas with variable size, not uniformly distributed, and/or to multiple punctate or linear non-shadowing densities.<br>3: A gross level of PIH, large round or confluent hypoechoic areas, and/or to linear densities, and/or to multiple cysts or multiple calcifications.<br>The overall score is the highest score in any of the 4 salivary glands.	2
Zhou et al. 2016a.	The same as Cornec et al. 2013.	2
Chen et al. 2016	0: Complete homogeneity.<br>1: Mild inhomogeneity.<br>2: Evident inhomogeneity.<br>3: Gross inhomogeneity.<br>The overall score is the highest score in any of the 4 salivary glands.	1
Qi et al.2017	0: Normal gland.<br>1: Slightly inhomogeneity.<br>2: Obvious rounded hypoechoic lesions.<br>3: Numerous and confluent hypoechoic lesions.<br>The highest score in any of the 4 salivary glands was the final score.	2

Su et al. 2004.	The same as Salaffi et al. 2008.	1
Yang et al. 2006.	The same as Salaffi et al. 2008.	2
Song et al. 2007.	The same as Salaffi et al. 2008.	1
Takagi et al. 2010.	A) 0 – 4 system:<br>0: Regular contour, no hypoechoic spots/areas, no echogenic bands.<br>1: Regular contour, small hypoechoic spots/areas, no echogenic bands.<br>2: Regular contour, round multiple hypoechoic spots/areas, no echogenic bands.<br>3: Irregular contour, round multiple hypoechoic spots/areas, presence of echogenic bands.<br>4: Irregular contour, irregular multiple hypoechoic spots/areas, presence of echogenic bands.<br>B) Quantitative characteristics: S value and SD value.	1
Cornec et al. 2013.	0: Normal homogeneous glands.<br>1: Small hypoechogenic areas without echogenic bands.<br>2: Multiple hypoechogenic areas measuring < 2 mm with echogenic bands.<br>3: Multiple hypoechogenic areas measuring 2 - 6 mm with hyperechogenic bands.<br>4: Multiple hypoechogenic areas measuring > 6 mm or multiple calcifications with echogenic bands.<br>The overall score is the highest score in any of the 4 salivary glands.	2
Hammenfors et al. 2015.	0: Hypo-/anechoic areas were not detected.<br>1: A few minor focal hypo-/anechoic areas that were considered within normal.<br>2: At least one of the glands was more severely affected with multiple focal hypo-/anechoic areas but some homogenous and normal appearing salivary gland tissue remained.<br>3: Severe generalised affection of at least two of the glands with minimal normal appearing glandular tissue remaining, as well as at least a grade 2 affection of the remaining gland(s).<br>0 - 1: were considered to correspond to normal/non-specific changes, and grade 2 - 3: to correspond to pathological changes.	2
Zhou et al. 2016b.	The grand was the same as Cornec et al. 2013.	2
**0 - 12 scoring system**		
Milic et al. 2010.	PIH was graded as 0 - 3 from homogeneous parenchyma to grossly inhomogeneous gland (mild parenchymal inhomogeneity, grade 1, was treated as a normal finding). A US inhomogeneity score was the sum of the grades for 4 salivary glands.	6
Lin et al. 2015.	The same as Milic et al. 2010.	6
**0 - 16 scoring system**		
Salaffi et al. 2008.	0: Normal gland.<br>1: Regular contour, small hypoechoi spots/areas without echogenic bands, regular or increased glandular volume (mean values 20 ± 3mm for the parotid glands and 13 ± 2mm for the submandibular glands), and ill defined posterior glandular border.<br>2: Regular contour, evident multiple scattered hypoechogenic areas usually of variable size (< 2mm) and not uniformly distributed, without echogenic bands, regular or increased glandular volume and ill defined posterior glandular border.<br>3: Irregular contour, multiple large circumscribed or confluent hypoechogenic areas (2 – 6 mm) and/or multiple cysts, with echogenic bands, regular or decreased glandular volume and posterior glandular border not visible.<br>4: Irregular contour, multiple large circumscribed or confluent hypoechogenic areas (> 6 mm) and/or multiple cysts or multiple calcifications, with echogenic bands, resulting in severe damage to the glandular architecture, decreased glandular volume, and posterior glandular border not visible.	6

	The overall score (0 - 16) is the sum of the score (0 - 4) of 4 salivary glands.	
Xu et al. 2010.	The same as Salaffi et al. 2008.	8
Milic et al. 2012.	The same as Salaffi et al. 2008.	7
Zhang et al. 2015.	The same as Salaffi et al. 2008.	7
Lin et al. 2015.	The same as Salaffi et al. 2008.	6
Qi et al.2017	0: Normal gland with homogeneous echogenicity.<br>1: There were a few confluent hypoechoic lesions.<br>2: Numerous and confluent hypoechoic lesions with diameter < 2 mm were present.<br>3: Numerous and confluent hypoechoic lesions with diameter 2 - 6 mm .<br>4: Numerous hypoechoic lesions with diameter > 6 mm.<br>Finally, the scores of the 4 glands were added as the final score.	5
**0 - 48 scoring system**		
Hocevar et al. 2005.	1) Parenchymal echogenicity was evaluated in comparison with the thyroid gland or when there was coincident thyroid gland disease by surrounding anatomical structures (muscular structures, subcutaneous fat). If the echogenicity was comparable to the thyroid, the grade was 0; if it was decreased, 1.<br>2) Homogeneity was graded 0- 3. 0: a homogeneous gland; 1: mild PIH; 2: evident PIH; 3: a gross PIH.<br>3) Hypoechogenic areals was graded 0– 3 (0: absent; 1: a few, scattered; 2: several; 3, numerous hypoechogenic areas).<br>4) Hyperechogenic reflections were graded 0 – 3 in the parotid glands (0: absent; 1: a few, scattered; 2: several; 3: numerous hyperechogenic reflections) and 0 (absent) – 1 (present) in the submandibular glands.<br>5) Clearness of salivary gland borders was graded 0 – 3 (0: clear, regular defined borders; 1: partly defined borders; 2: ill-defined borders; 3: borders not visible).<br>The overall score is the sum of the above five parameters for the 4 salivary glands.	17
Milic et al. 2009.	The same as Hocevar et al. 2005.	19
Zhang et al. 2015.	The same as Hocevar et al. 2005.	15
Lin et al. 2015.	The same as Hocevar et al. 2005.	17
Kong et al. 2011.	1) Homogeneity was graded 0 – 3.<br>2) Parenchymal echogeneity was graded 0 – 3.<br>3) The presence of hypoechogenic areas was graded 0 – 3.<br>4) Clearness of salivary gland borders was graded 0 – 3.<br>The overall score is the sum of the score for the 4 salivary glands.	un-known

Reference

Baldini C. *et al*. Salivary gland ultrasonography: a highly specific tool for the early diagnosis of primary Sjögren's syndrome. Arthritis Res Ther. 17:146 (2015).

Chen S. *et al*. Combination of Salivary Gland Ultrasonography and Virtual Touch Quantification for Diagnosis of Sjögren’s Syndrome: A Preliminary Study. BioMed Res Int. 2016:2793898 (2016).

Cornec D. *et al*. Contribution of salivary gland ultrasonography to the diagnosis of Sjögren's syndrome: toward new diagnostic criteria? Arthritis Rheum. 65:216-225 (2013).

El Miedany Y.M. *et al*. Quantitative ultrasonography and magnetic resonance imaging of the parotid gland: can they replace the histopathologic studies in patients with Sjögren's syndrome? Joint Bone Spine. 71:29-38 (2004).

Hammenfors D.S. *et al*. Diagnostic utility of major salivary gland ultrasonography in primary Sjögren's syndrome. Clin Exp Rheumatol. 33:56-62 (2015).

Hocevar A. *et al*. Ultrasonographic changes of major salivary glands in primary Sjögren's syndrome. Diagnostic value of a novel scoring system. Rheumatol. 44:768-772. (2005).

Kong L.H. *et al*. The comparative study of salivary gland scintigraphy quantitative analysis and high frequency ultrasound and labial gland biopsy in patients with Sjögren's syndrome. Med J of Commun. 25:133-136 (2011).
Lin D.F. *et al*. Cross-sectional comparison of ultrasonography scoring systems for primary Sjögren's syndrome. Int J Clin Exp Med. 8:19065-19071 (2015).
Milic V.D. *et al*. Diagnostic value of salivary gland ultrasonographic scoring system in primary Sjögren's syndrome: a comparison with scintigraphy and biopsy. J Rheumatol. 36:1495-1500 (2009).
Milic V.D. *et al*. Major salivary gland sonography in Sjögren's syndrome: diagnostic value of a novel ultrasonography score (0-12) for parenchymal inhomogeneity. Scand J Rheumatol. 39:160-166 (2010).
Milic V. *et al*. Ultrasonography of major salivary glands could be an alternative tool to sialoscintigraphy in the American-European classification criteria for primary Sjögren's syndrome. Rheumatol. 51:1081-1085 (2012).
Niemela R.K. *et al*. Ultrasonography of salivary glands in primary Sjögren's syndrome. A comparison with magnetic resonance imaging and magnetic resonance sialography of parotid glands. Rheumatol. 43:875-879 (2004).
Qi X. *et al*. Comparison of the diagnostic value of four scoring systems in primary sjögren's syndrome patients. Immunol Lett. 188:9-12 (2017).
Salaffi F. *et al*. Ultrasonography of salivary glands in primary Sjögren's syndrome: a comparison with contrast sialography and scintigraphy. Rheumatol. 47:1244-1249 (2008).
Song S.J. *et al*. Comparative study on parotid ultrasonography and scintigraphy and labial gland biopsy in Sjögren's syndrome. Chin J Med Ultrasound. 4:159-161 (2007).
Su Y.J, Du L.F. & Shi L.L. Comparative study on parotid ultrasonography and sialography in Sjögren's syndrome. Chin J Ultrasonogr. 13:588-590 (2004).
Takagi Y. *et al*. Salivary gland ultrasonography: can it be an alternative to sialography as an imaging modality for Sjögren's syndrome? Ann Rheum Dis. 69:1321-1324 (2010).
Theander E. & Mandl T. Primary Sjögren's syndrome: The diagnostic and prognostic value of salivary gland ultrasonography using a simplified scoring system. Arthritis Care Res. 66:1102-1107 (2014).
Xu Z.H. *et al*. Value of sonographic score in the diagnosis of salivary gland involvement in patients with Sjögren's syndrome. Chin J Ultrasonogr. 19:977-980 (2010).
Yang B. *et al*. Comparative study on salivary gland SPECT and ultrasonography in diagnosis of Sjögren's syndrome. China Medical Herald. 3:148-149 (2006).
Zhang X. *et al*. Ultrasonographic evaluation of major salivary glands in primary Sjögren's syndrome: comparison of two scoring systems. Rheumatol. 54:1680-1687 (2015).
Zhou M. *et al*. Study on the value of salivary gland ultrasonography in Sjögren's syndrome. Chin J Rheumatol. 20:317-320 (2016a).
Zhou M. *et al*. The comparative study of diagnositic value for primary Sjögren's syndrome between salivary gland ultrasonography and scintigraphy. J Clin Exp Med. 15:87-90 (2016b).
